# A deeper look into the functions of right ventricle using three-dimensional echocardiography: the forgotten ventricle in children with systemic lupus erythematosus

**DOI:** 10.1007/s00431-023-04936-y

**Published:** 2023-04-11

**Authors:** Shaimaa Rakha, Ayman Hammad, Hala Elmarsafawy, Mai S. Korkor, Riham Eid

**Affiliations:** 1 Pediatric Cardiology Unit, Mansoura University Children’s Hospital, Faculty of Medicine, Mansoura University, Mansoura, Egypt; 2 Pediatric Nephrology Unit, Mansoura University Children’s Hospital, Faculty of Medicine, Mansoura University, Mansoura, Egypt

**Keywords:** Systemic lupus erythematosus, Right ventricle, 3D echocardiography

## Abstract

Studies on the right ventricular dysfunction (RV) in systemic lupus erythematosus (SLE) patients are limited, particularly in the pediatric age group. The study aimed to identify subclinical RV alterations in childhood-onset SLE (c-SLE) using conventional and three-dimensional echocardiography (3DE). Forty SLE pediatric patients and 40 healthy controls were included. Disease activity and chronicity were evaluated by SLE disease activity index (SLEDAI) score and SLE damage index (SDI). Participants underwent detailed RV echocardiographic examination with conventional and 3DE assessment using 3D auto RV software. Patients included 35/40 (87.5%) females with mean age of 15.6 ± 1.7 years. Using conventional pulmonary artery systolic pressure echocardiography-derived measurement, none of the c-SLE patients had pulmonary hypertension. By 3DE, RV end-systolic and end-diastolic volumes (*p* = < 0.001, 0.02, respectively) were greater, whereas 3D-derived RV ejection fraction (*p* < 0.001), septal, and lateral longitudinal strain (both *p* < 0.001) were lower in SLE. SDI displayed a significant correlation with 3D auto RV ejection fraction (EF), tricuspid annular plane systolic excursion (TAPSE), fractional area change, and RV longitudinal strain (RVLS)-free wall (*p* = 0.01, 0.003, 0.007, and < 0.001, respectively). Cumulative SLEDAI score also showed a significant correlation with RV EF, TAPSE, FAC, and RVLS-free wall (*p* = 0.03, 0.007, 0.002, and < 0.001, respectively). By multivariate regression analysis, SDI remained an independent predictor of RVLS-free wall (ß coefficient − 0.4, *p* = 0.03) and TAPSE (ß − 0.5, *p* = 0.02).

*  Conclusion*: Subtle right ventricular myocardial dysfunction could be detected in childhood-onset SLE patients, especially via 3D-derived auto RV echocardiographic parameters, despite the absence of evident pulmonary hypertension. These parameters correlate with the SLE disease activity and chronicity scores.

**Table:** 

**What is Known:**
•*Diseases of the cardiovascular system are one of the most common causes of morbidity and mortality in SLE patients.*
•*RV labeled the forgotten ventricle in many diseases, was also forgotten in SLE patients and has been rarely addressed in adults, with scarce research in pediatrics.*
**What is New:**
•*Right ventricular functions are affected in children with SLE in comparison to healthy controls, especially three-dimensional echocardiography-derived parameters, which is an aspect that has not been investigated in previous research in the pediatric age group.*
•*Some of the detected myocardial dysfunctions of the right ventricle correlated with SLE disease activity and chronicity-related scores.*

## Introduction

Systemic lupus erythematosus (SLE) is a systemic autoimmune disorder associated with chronic inflammation and immune complex deposition in involved organs. Cardiovascular diseases are considered one of SLE patients’ most common causes of morbidity and mortality [1–3]. Moreover, SLE patients have a significantly higher risk of developing heart failure, coronary artery disease, myocardial infarction, myocarditis, pericarditis, conduction system disease, and valvular disease [4–7]. In addition, several studies have proved subclinical left ventricular (LV) involvement in SLE patients without a prior history of cardiac disease in the early SLE stages, such as LV hypertrophy, systolic dysfunction, and LV diastolic dysfunction [2, 8]. However, the right ventricle (RV), which is labeled the forgotten ventricle in many diseases [9, 10], was also overlooked in SLE patients and has been rarely addressed in adults, with scarce research in pediatrics [11–13].

The two-dimensional (2D) approach to the RV via conventional echocardiography (ECHO) is technically challenging compared to the one of the LV. This is because of its complex triangular crescentic shell anatomy. Moreover, the RV location retrosternal forward to the LV add to the challenge of its 2D assessment [14]. Therefore, the 2D-derived parameters for RV functions do not precisely represent the global RV functions [15].

Three-dimensional echocardiography (3DE) overcomes complex geometry problems, thus enabling a comprehensive assessment of RV chambers, internal volume, and RV ejection fraction (EF) determination. It may be considered an alternative to cardiac magnetic resonance imaging (cMRI) [16]. Further advancement of 3D speckle tracking echocardiography (STE) now allows the assessment of RV longitudinal, circumferential, and radial deformation, with the superiority of longitudinal strain in RV contraction [17]*.*

As far as we know, a single study in the adult population has investigated RV function using 3DE parameters in SLE patients [18]. However, in the pediatric age group, this feature of cardiac function in SLE patients has never been addressed. Therefore, this study aimed to evaluate whether the subclinical RV function alterations could be detected using 3DE in pediatric patients with childhood-onset SLE (c-SLE) compared to a healthy control group.

## Materials and methods

The prospective observational case–control study included 40 pediatric patients with SLE and 40 healthy controls. The work was conducted between November 2020 and February 2022 after approval of the institutional research board (IRB) of Mansoura University, Faculty of Medicine, Egypt. Informed consent was obtained from legal guardians for all study participants. The cases were recruited from the SLE outpatient clinic in a single tertiary center, Mansoura University Children’s Hospital, Egypt.

### Inclusion criteria

Pediatric patients up to 18 years old with SLE having a duration of illness of at least 3 years were included, in addition to age and sex-matched healthy controls for comparison purposes. All included patients diagnosed after fulfilling the American College of Rheumatology (ACR) classification criteria for SLE [19]. In addition, all included patients had normal kidney function defined as an estimated glomerular filtration rate above 90 ml/min/1.73 m^2^ (calculated by Schwartz formula) [20].

### Exclusion criteria

Patients with congenital heart disease, arrhythmia, primary cardiomyopathies, or significant cardiac valve disorders (more than mild stenosis or regurgitation) were excluded. Besides, we excluded patients with acute hemodynamic instability (e.g., overt heart failure, significant pericardial effusion). Additionally, cases with chronic obstructive pulmonary disease, interstitial lung disease, and other restrictive pulmonary abnormalities were excluded. Moreover, we did not include patients with impaired kidney function, uncontrolled hypertension, or any other systemic disease well-known to impair cardiac function or cases with inadequate ECHO image quality that interfere with the 3D analysis.

### Methods

Detailed history, including the patient’s age, duration of SLE, and medications were obtained. Blood samples were taken for ESR as an inflammatory marker and autoantibodies assay (antidsDNA) in addition to complete blood count (CBC), complement 3 (C3), urinalysis, and a 24-h urinary protein assessment to incorporate into disease activity score. Current and previously reported disease activity (evaluated using the Systemic Lupus Erythematosus Disease Activity Index 2000 (SLEDAI-2 K) was documented [21]. The cumulative SLEDAI score is calculated as a mean of all reported SLEDAI scores for each patient during the follow-up period. The possible results define four activity categories as follows: no disease activity (SLEDAI = 0), mild activity (SLEDAI = 1–5), moderate activity (SLEDAI = 6–10), high activity (SLEDAI = 11–19), and very high activity (SLEDAI ≥ 20). In addition, disease-related damage evaluated by Systemic Lupus International Collaborating Clinics/American College of Rheumatology Damage Index (SDI) was also obtained [22].

#### Anthropometrics and vitals

Body weight (in kg), height (in cm), body mass index (BMI in kg/m^2^), and surface area (in m^2^) were assessed at the time of ECHO. Blood pressure (systolic and diastolic) in mmHg and heart rate (beat/minute) were evaluated before starting the ECHO.

#### Echocardiographic assessment

Transthoracic ECHO recordings from all the children were performed using a standard ECHO machine EPIQ CVx Release 5.0 (Philips Medical Systems, Bothell, WA, USA 2018) equipped with an X5-1 matrix array transducer (5–1 MHz).

**Fig. 1:**
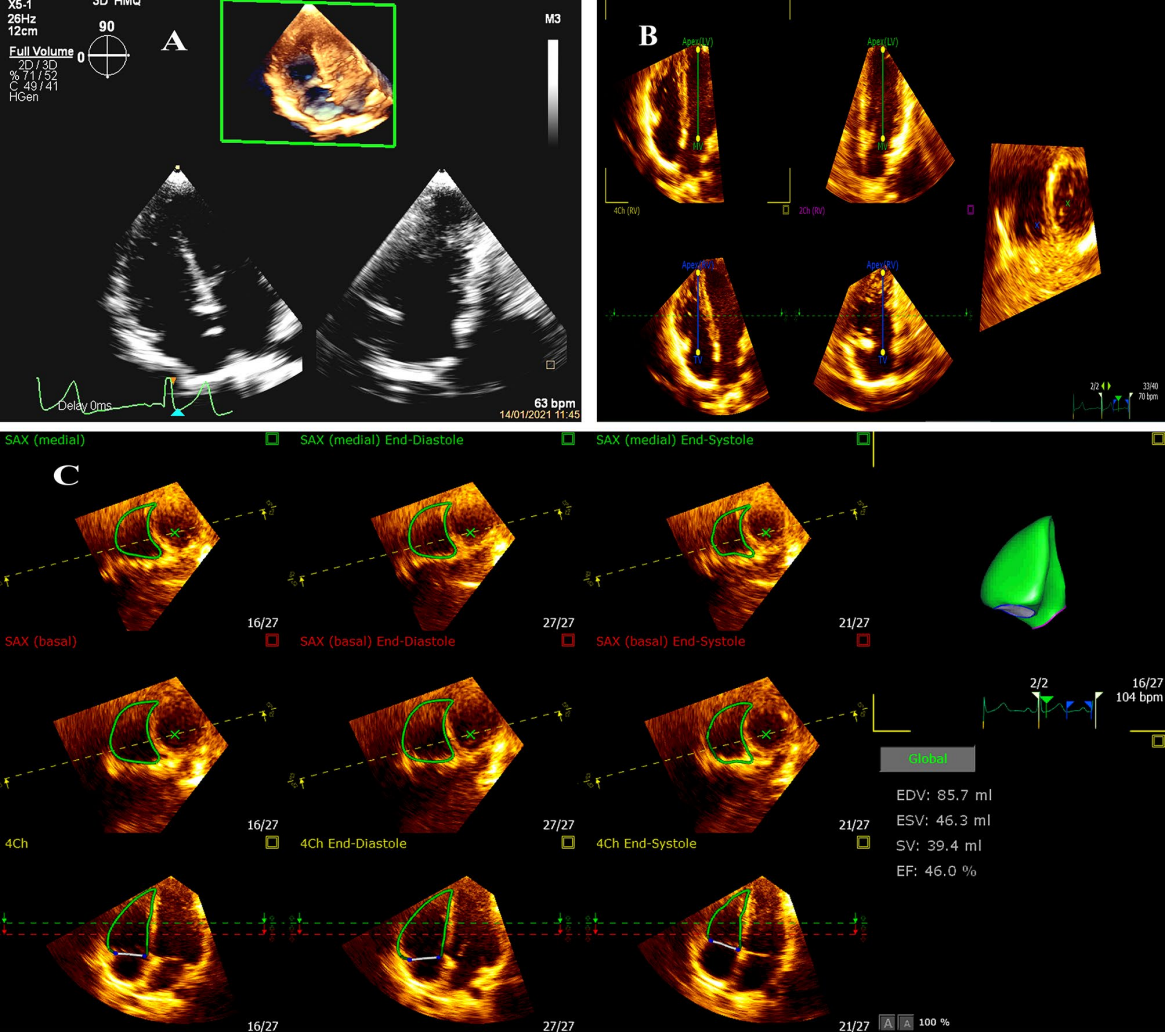
The fully automated right ventricular (RV) functional assessment. **A** The RV focus three-dimensional echocardiography (3DE) full-volume data set acquisition. **B** The software adjusts the five landmarks automatically, which are adjusted as needed. **C** Automatic tracking of the RV border at the end diastole and end systole and the results of SLE patient showing 3D-derived RV EF 46%

1. Conventional ECHO: Standard transthoracic ECHO, including apical four-chamber, three-chamber, two-chamber, parasternal short-axis and long-axis views, as well as M. mode, conventional Doppler, and tissue Doppler evaluation, were performed based on the recommendations of the American Society of Echocardiography [23]. M-mode images were obtained from two-dimensional images with parasternal views. In both systole and diastole, interventricular septal thicknesses (IVST), LV posterior wall diameter (LVPWD), and internal LV diameter were measured in mm and z-score. Z-scores for M.mode-derived measurements were assessed according to the surface area of the patients based on Kampmann et al. z-scores [24]. In addition, LV mass index (g/m^2^) and LV systolic functions, including fraction shortening (FS) and ejection fraction (EF) in percentage, were measured. Color flow Doppler was assessed across the cardiac valves to estimate the presence and degree of regurgitation across tricuspid and pulmonary valves. Pulsed wave Doppler was performed across cardiac valves, including pulmonary and tricuspid peak velocities, early diastolic flow (E-wave), late diastolic flow (A-wave) velocities in centimeter/second, and the E: A ratio was calculated for tricuspid valve. The pulmonary artery systolic pressure (PASP) was calculated in mmHg as the sum of both the tricuspid regurgitation pressure gradient (using the Bernoulli equation from tricuspid regurgitation peak velocity) and the RA pressure (assumed according to IVC diameter and respiratory-related collapsibility). Pulmonary hypertension (PH) was considered when PASP > 30 mmHg at rest [25].
2. Pulsed-tissue Doppler imaging: Early diastolic (E′ wave), late diastolic (A′ wave), and systolic (S′ wave) velocities were measured at the lateral parts of the tricuspid annulus on the apical four-chamber views by pulsed-wave tissue Doppler (all in centimeters per second), then E/E′ ratio, was calculated. The tissue Doppler-derived-Tei index of RV was calculated using the following formula: IVCT′ + IVRT′/ET′, where isovolumic contraction time (IVCT') from the A′ wave end to S′ wave beginning, while isovolumic relaxation time (IVRT') was calculated as the time from the S′ wave end to E′ wave beginning, and ET is the ejection time from the beginning to the end of S′ wave.
3. Three-dimensional auto RV: A 3DE full-volume ECG-gated dataset at RV-focused apical four-chamber view was acquired using Heart Model Acquisition mode (HM ACQ) at a frame rate of more than 20–25 as recommended by ASE [26]. RV full-volume 3DE data sets were analyzed by a fully automated 3D RV quantification software (3D auto RV, Philips Healthcare). The software automatically detected the RV endocardial border using artificial intelligence, consisting of initial RV orientation, global shape recognition, and 3D speckle tracking throughout one cardiac cycle. ECG-gated acquisition of heartbeats was mandatory for 3D auto RV to automatically define the end-diastolic and end-systolic time points for frame tracking to allow the analysis of the 3D data; otherwise, the software would not yield functional results. The software allows automated contour detection of the right ventricular short axis and four-chamber views with automatic results display (see Fig. 1). The software analyzes a single beat at a time; moreover, it allows choosing the beat with the most accurate tracking to demonstrate its results. 3D Auto RV maintains the functionality of the TOMTEC 4D RV-FUNCTION software, including view adjustment and tracking revision. The software automatically generates the following 3D Echo-derived parameters for RV: end diastolic volume (EDV) in mL, end systolic volume (ESV) in mL, end-diastolic volume indexed to surface area (EDVi) in (mL/m2), end-systolic volume indexed to surface area (ESVi) in (mL/m2), EF in percentage and stroke volume (SV) in mL, fractional area change of right ventricle (FAC) in percentage, tricuspid annular plane systolic excursion (TAPSE) in mm, and RV longitudinal strain (RVLS) in percentage at the septum and free wall.

### Study power

The sample was sized using G*Power 3.1.9.2 software to provide a power of 99% and an alpha error of 0.05. The calculation was based on the mean and standard deviation (SD) of 3D-derived RVEF results of Buonauro et al. [18] on an adult population with SLE. The calculated sample was at least 31 in each of the cases and control group.

### Reproducibility

The 3D auto RV reproducibility was determined through intraobserver and interobserver intraclass correlation coefficient (ICC) evaluation for the 3D auto RV-derived RV EF. In a randomly selected sample of 15 patients, the same observer performed an offline reassessment of 3D-derived RV EF a week later for intraobserver variability determination. In contrast, for interobserver variability, the same patients were examined by another observer blinded to the initial results.

### Statistical analysis

Statistical analysis was performed using the Statistical Package for the Social Sciences (SPSS) (SPSS, Inc, an IBM Company, Chicago, IL, USA) version 25. Shapiro–Wilk test was used to test data normality of distribution. Data were presented as mean ± standard deviation (SD) or median whenever appropriate. The chi-square or Fisher’s exact test was used to compare categorical variables. When relevant, continuous variables were compared using the Student’s *t*-test or Mann–Whitney *U* test. If the *p*-value < 0.05, it will be considered statistically significant. Linear correlations between the variables were evaluated using the Pearson correlation coefficient. Multivariate regression analysis was performed to assess the. Intra- and inter-observer reproducibility was quantified using the intraclass correlation coefficient (ICC).

## Results

### Participants and descriptive data

Between November 2020 and February 2022, 40 pediatric patients with SLE and 40 matched healthy controls were enrolled in the study. The demographic characteristics and clinical data of the study groups are summarized in Table 1. Patients and controls had matching age and gender distribution, while BMI and surface area were significantly higher in patients than in controls. The age range of the study cases was 10–17.5 years.

**Table 1:** Demographic characteristics and clinical data of the study groups

**Variables**		Cases*	Controls*	*p*-value**
		**(*N* = 40)**	**(*N* = 40)**	
**Gender** (M/F)		5/35	7/33	0.5
**Age** (years)		15.6 ± 1.7	14.9 ± 1.6	0.06
**Body mass index **(kg/m^2^)		24.2 ± 3.9	21.3 ± 2.6	< 0.001
Classification of weight according to BMI	Normal weight	22 (55%)	36 (90%)	
	Overweight	11 (27.5%)	4 (10%)	
	Obese	7 (17.5%)	0 (0%)	
**Surface area** (m^2^)		1.6 ± 0.19	1.4 ± 0.18	< 0.001
**Systolic blood pressure** (mmHg)		110.8 ± 7.5	108.3 ± 7.2	0.11
**Diastolic blood pressure** (mmHg)		71.8 ± 7.3	69.5 ± 7.2	0.17
**Heart rate** (bpm)		85.4 ± 14.8	79.6 ± 7.1	0.03

*M* male, *F* female, *bpm* beat per minute

*Data are presented as mean ± SD or number (percentage) or ratio

**Statistically significant difference if (*p* < 0.05)

Table 2 demonstrates the clinical and laboratory data of included SLE patients. The mean age at initial SLE diagnosis was 12.8 ± 2.9 years. Mean SLEDAI at the time of ECHO was 6 ± 5.6 with 19 patients (47.5%) had SLEDAI score > 5 and mean cumulative SLEDAI 8.3 ± 5.2, cumulative SLEDAI > 5 in 26 (65%), while 17 (42.5%) had SDI score ≥ 1.

**Table 2:** The clinical and laboratory data of included SLE patients

**Variables**	Results*
**Age at SLE diagnosis **(years)(mean ± SD)	12.8 ± 2.9
**Duration of illness **(months) (mean ± SD)	52.2 ± 14.6
**Clinical manifestations**: *N* **(%)**	
• Nephritis	21 (52.5%)
• Arthritis	25 (62.5%)
• Serositis	18 (45%)
• Mucocutaneous manifestations	30 (75%)
• CNS involvement	7 (17.5%)
**SLEDAI score **(at time of ECHO) (mean ± SD)	6 ± 5.6
• Mild activity	• 21 (52.5%)
• Moderate activity	• 14 (35%)
• High activity	• 4 (10%)
• Very high activity	• 1 (2.5%)
**Cumulative SLEDAI** (mean ± SD)	8.3 ± 5.2
**SDI** (mean ± SD)	0.88 ± 1.3
**Anti-dsDNA** IU/mL (at time of ECHO) (mean ± SD)	120 (0–560)
**ESR** (mm/h) (mean ± SD)	74.6 ± 41.2
**Cyclophosphamide therapy** **Mycophenolate mofetil** **Azathioprine**	17 (42.5%) 20 (50%) 8 (20%)

*CNS* central nervous system, *ECHO* echocardiography, *ESR* erythrocyte sedimentation rate, *SD* standard deviation, *SDI* systemic lupus damage index, *SLEDAI* systemic lupus erythematosus disease activity index

*Data are presented as mean ± SD or median (interquartile range), or number (%)

### Main results: ECHO parameters of patients compared to controls

The conventional ECHO assessment of the two study groups is presented in Table 3. Notably, RVAW, LVPW, IVS thickness, and z-scores were significantly higher in patients than in controls. Moreover, the LV mass index was also significantly higher in SLE cases than in the healthy participants, with nonsignificant lower ejection fraction and fractional shortening in the c-SLE patients. Regarding color flow, 70% of cases had trivial or mild tricuspid regurgitation; however, 32% of controls had trivial tricuspid valve regurgitation. In the SLE group, pulmonary regurgitation was detected in 17.5% of cases, with 7.5% mild to moderate in degree, while in the control group, trivial regurgitation was detected only in 2 cases (5%). None of our patients had pulmonary hypertension based on PASP.

**Table 3:** The conventional echocardiographic characteristics of the two study groups

	**Parameters**		Cases*	Controls*	*p*-value**
			**(*N*=40)**	(***N***=40)	
**M. Mode**	RVAWd	mm	4.8±1.1	3.7±0.7	<0.001
		Z-score	2.9±1.8	1.1±1.2	<0.001
	RVIDd	mm	15±5.1	14±4.6	0.3
		Z-score	-0.29± 1.6	-0.28±1.7	0.9
	IVST systole	mm	8.6±1.9	7.2±1.3	<0.001
		Z-score	-0.54±0.94	-1.2±0.67	0.001
	IVST diastole	mm	6.7±1.4	5.6±0.8	<0.001
		Z-score	-0.87±1.1	-1.4±0.77	0.003
	LV diameter in systole	mm	32±6.9	28±3.8	0.001
		Z-score	0.89±2.2	-0.08±1.3	0.019
	LV diameter in diastole	mm Z-score	49±6.7	45±4	<0.001
			0.95±1.9	0.15±1.2	0.03
	LVPWD systole	mm	8.5(4.9-12.3)	8.9(7.3-10)	0.4
		Z-score	-2.3(-4.4-0.16)	-1.7(-3.1- -1.2)	0.005
	LVPWD diastole	mm	6.7(3.9-10.4)	5.6(4.3-7.8)	<0.001
		Z-score	-0.62(-2.3-1.96)	-1.04(-2.1- -0.4)	<0.001
	LV mass index	(g/m^2^)	68.6 ±21.4	52.8±12.4	<0.001
	LV mass	(g)	111.2±38.5	67.4±22.1	<0.001
	LV EF	(%)	64.03±7.9	66.7±4.7	0.063
	LV FS	(%)	34.8±6.1	36.5±3.3	0.13
**Color flow**	Tricuspid. regurgitation	No	12(30%)	27(67.5%)	<0.001
		Trivial	15(37.5%)	13(32.5%)	
		Mild	13(32.5%)	0	
	Pulmonary regurgitation	No	33(82.5%)	38(95%)	0.26
		Trivial	4(10%)	2(5%)	
		Mild	2(5%)	0	
		Moderate	1(2.5%)	0	

*RVAW* right ventricle anterior wall, *d* diastole, *RVID* right ventricle internal diameter, *IVST* interventricular septal thickness, *LVPWD* left ventricle posterior wall diameter, *LV* left ventricle, *EF* ejection fraction, *FS* fraction shortening

*Data are presented as mean ± SD or median, IQR (inter-quartile range)

**Statistically significant difference if (*p* < 0.05)

In Table 4, RV pulsed and tissue Doppler imaging parameters are demonstrated in cases compared to controls. A significantly higher mean of tricuspid A wave and consequently lower E/A ratio was confirmed in cases than in controls. Furthermore, statistically significantly lower mean S′ velocity in the patients’ cohort than the controls, with an increase significantly in Tei index and E/E′ in the SLE group compared to the normal children (*p* = < 0.001, 0.002, respectively).

**Table 4:** Right ventricular Doppler parameters in cases and controls

**Parameters**		Cases*	Controls*	*p*-value**
		**(*N*** **= 40)**	**(*N* = 40)**	
**Conventional pulsed Doppler**	Tricuspid E (cm/s)	67.3 ± 11.8	68.2 ± 10.2	0.7
	Tricuspid A (cm/s)	55.6 ± 13.9	46.5 ± 5.1	< 0.001
	Tricuspid E/A ratio	1.3 ± 0.33	1.47 ± 0.17	0.001
	Pulmonary valve (cm/s)	86.3 (57–129)	83.9 (64.5–110)	0.7
	Tricuspid regurgitation	(*N* = 28)	(*N* = 13)	
	–TR velocity (m/s)	1.9 ± 0.3	1.7 ± 0.17	0.049
	-Mean PASP(mmHg)	25.8 ± 4.6	18.2 ± 2.9	< 0.001
**Tissue Doppler**	RV E′ (cm)	13.9 (7–19.3)	15 (11.4–19.4)	0.004
	RV A′ (cm)	11.6 ± 4.2	10.6 ± 1.3	0.15
	RV S′ (cm)	12.1 ± 1.5	13.3 ± 1.7	0.002
	RV Tei index	0.59 ± 0.1	0.46 ± 0.04	< 0.001
	E/E′	5.2 ± 1.2	4.6 ± 0.69	0.002

*PASP* pulmonary artery systolic pressure

*Data are presented as mean ± SD or median IQR (inter-quartile range)

** Statistically significant difference if (p < 0.05)

Table 5 presents 3D auto RV-acquired data in patients compared to controls. The 3D-derived RV EF was significantly lower in the SLE group compared to the control group, with *p* < 0.001. Auto RV-derived lateral and septal LS were impaired considerably in SLE compared to controls (*p* < 0.001). Additionally, RV TAPSE and FAC were substantially lower in patients vs. controls (*p* = 0.001, < 0.001, respectively).

**Table 5:** Right ventricular 3D auto RV acquired data

**Parameters**	Cases* (*N* = 40)	Controls* (*N* = 40)	*p*-value**
EDV (mL)	101.8 ± 31.4	83.1 ± 18.6	0.002
EDVi (mL/m2)	62.8 ± 16.1	63.3 ± 8.9	0.9
ESV (mL)	50.5 ± 22.9	33.2 ± 8.9	< 0.001
ESVi (mL/m2)	31.2 ± 12.1	25.1 ± 4.4	0.004
SV (mL)	51.3 ± 13.5	49.9 ± 10.5	0.6
EF (%)	51.5 ± 8.2	60.4 ± 3.7	< 0.001
RVDd-base (mm)	39.3 ± 5.5	37.1 ± 3.6	0.04
RVDd-mid (mm)	35.8 ± 6.5	33.4 ± 4.2	0.07
RVLd (mm)	73.5 ± 7.4	68.3 ± 5.2	0.001
TAPSE (mm)	19.9 ± 4.6	23.9 ± 2.4	0.001
FAC (%)	45.2 ± 8.2	52.3 ± 4.5	< 0.001
RVLS-septum (%)	−19.7 ± 4.9	−23.4 ± 4.6	< 0.001
RVLS-free wall (%)	−27.1 ± 5.7	−34.1 ± 3.1	< 0.001

*EDV* end-diastolic volume, *EDVi* end-diastolic volume index, *ESV* end-systolic volume, *ESVi *end-systolic volume index*, SV* stoke volume, *EF* ejection fraction, *RVDd* right ventricle dimension at end-diastole, *RVLd* right ventricle longitudinal diameter, *TAPSE* tricuspid annular plane systolic excursion, *FAC* fractional area change, *RVLS* right ventricular longitudinal strain

*Data are presented as mean ± SD

**Statistically significant difference if (*p* < 0.05)

### Secondary results

Regarding SLE patients, no significant difference was found between male and female subjects, patients with and without nephritis, arthritis, serositis, cutaneous, and CNS manifestations (*p* > 0.05 for all). Also, no significant difference was found between those who received or did not receive cyclophosphamide, mycophenolate mofetil, or azathioprine.

**Fig. 2:**
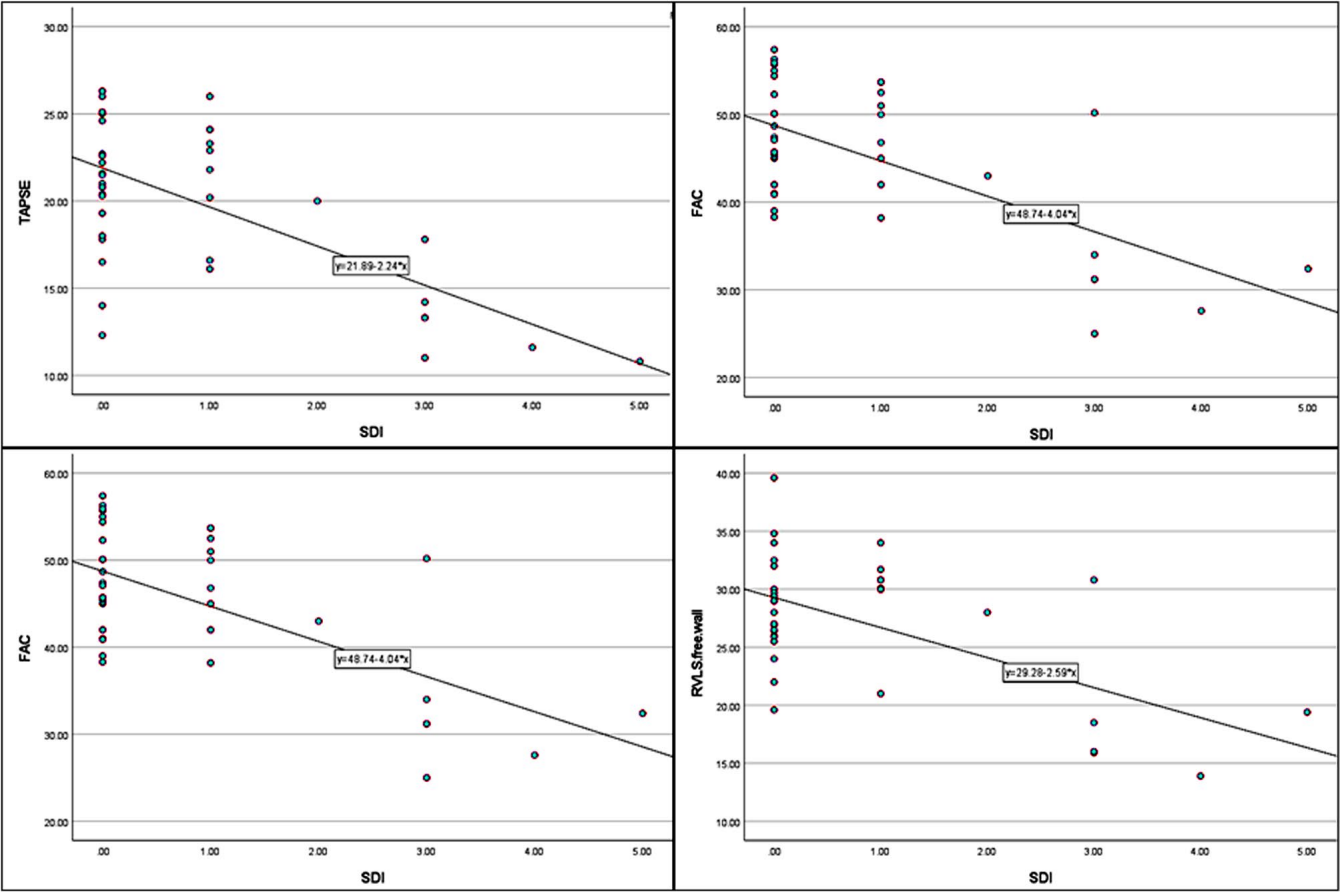
Scatterplot and regression lines of individual values of SDI (*x*-line) and corresponding values (*y*-line) of 3D auto RV derived-EF (ejection fraction), TAPSE (tricuspid annular plane systolic excursion), FAC (fractional area change), and RVLS free wall (right ventricle longitudinal strain)

The correlations of the patient’s age, disease activity, and damage indices with RV ECHO parameters are shown in Table 6. SLEDAI score at the time of ECHO correlated to RVLS (free wall) (*r* = 0.4, *p* = 0.01), while cumulative SLEDAI score negatively correlated with EF, TAPSE, and FAC (*r* = − 0.3, − 0.4, − 0.5, − 0.5; *p* = 0.03, 0.007, 0.002, < 0.001, respectively). SDI negatively correlated with EF, TAPSE, FAC, and RVLS free wall (Fig. 2). TASPE also correlated significantly with RVEF (*r* = 0.7, *p* = < 0.001). However, the duration of illness and anti-ds DNA level was not significantly correlated with the studied echocardiographic parameters. By multivariate regression analysis, SDI remained an independent predictor of RVLS-free wall (ß coefficient − 0.4, *p* = 0.03) and TAPSE (ß − 0.5, *p* = 0.02).

**Table 6:** Correlations of patients’ age, activity, and chronicity indices of SLE with RV echocardiographic functional parameters

**Echocardiography**		**Age at time of ECHO**		**SLEDAI at time of ECHO**		**Cumulative SLEDAI**		**Damage index**	
**Modality**	**Parameters**	r*	P**	r*	P**	r*	P**	r*	P**
**Conventional ECHO**	RVAWd z-score	−0.1	0.5	0.09	0.6	0.2	0.2	0.4	0.02
	Tricuspid E/A ratio	0.2	0.1	0.08	0.6	−0.2	0.3	− 0.3	0.04
	RV Tei index	−0.03	0.9	0.03	0.8	0.09	0.6	−0.03	0.8
	RV E/ E′	0.04	0.8	−0.05	0.8	0.009	0.9	0.004	0.9
**3D auto RV**	EDV	0.3	0.1	0.01	0.9	0.3	0.1	0.2	0.3
	EDVi	0.1	0.5	0.2	0.3	0.3	0.09	0.2	0.4
	ESV	0.3	0.08	− 0.04	0.8	0.4	0.06	0.4	0.02
	ESVi	0.2	0.3	0.09	0.6	0.4	0.02	0.3	0.06
	3D-derived EF	−0.2	0.1	0.05	0.8	−0.3	0.03	−0.4	0.01
	RVLd	0.2	0.2	0.07	0.6	0.2	0.2	0.3	0.03
	TAPSE	0.1	0.5	−0.01	0.9	−0.4	0.007	−0.5	0.003
	FAC	−0.2	0.3	0.02	0.9	−0.5	0.002	−0.4	0.007
	RVLS-septum	0.3	0.1	−0.2	0.2	0.04	0.8	−0.02	0.8
	RVLS-free wall	−0.05	0.7	0.4	**0.01**	−0.5	< 0.001	−0.6	< 0.001

*EDV* end-diastolic volume, *EDVi* end-diastolic volume index, *EF* ejection fraction, *ESV* end systolic volume, *ESVi* end systolic volume index, *SV* stoke volume, *TAPSE* tricuspid annular plane systolic excursion, *FAC* fractional area change, *RV* right ventricle, *RVAWd* right ventricle anterior wall diameter in diastole, *RVLS* right ventricular longitudinal strain

*r: Pearson’s correlation coefficient

**P: Statistically significant difference if (*p* < 0.05)

Intraobserver variability ICC for 3D-derived RV EF was 0.96, while ICC for interobserver variability of the corresponding parameter was 0.94.

## Discussion

There have been noticeable improvements in the survival of c-SLE [27, 28]; however, children and adolescents with SLE are now faced with considerable morbidity resulting from sequelae of disease activity and medication side effects. Hence, the need for monitoring irretrievable organ damage to help provide the best care. Furthermore, most of the research in c-SLE has focused on LV functions, even on using 3DE [29, 30]. Therefore, the current work shed light on the subclinical myocardial functional alterations of the forgotten ventricle “the RV” in asymptomatic pediatric patients with c-SLE in relation to controls using conventional ECHO, tissue Doppler, and specific emphasis on 3DE-derived parameters. In addition, the disease’s long-term impact is evidenced by SDI and cumulative SLEDAI score.

Although, the mean heart rate was significantly higher in the patients’ group than in the control group, it was within the average heart rate range for age. None of the included cases could be described with sinus tachycardia diagnosis; therefore, it was not expected to affect cardiac function. Similar findings were reported in a study from Turkey on juvenile SLE [31]. Nevertheless, several studies documented sinus tachycardia in SLE patients, especially in the adult population. Sinus tachycardia was reported in 18% of Myung et al. SLE cases. They suggested autonomic nervous system involvement as a possible etiology [32]. Guzman et al. identified sinus tachycardia in 50% of the patients with SLE and correlated it to the Mexican SLEDAI score of activity [33]. Utset et al. reported chronic tachycardia in 14.8% of their SLE cohort. On multivariate regression, SLEDAI score, younger age, and poor physical function score independently correlated with chronic tachycardia [34].

In the current study, although tricuspid regurgitation in the c-SLE cases was documented in a higher percentage than in the controls with significantly higher PASP, none of our cases had pulmonary arterial hypertension (PAH). Adrovic et al. reported a consistent finding of increased PASP in SLE cases; nevertheless, they documented only one patient with juvenile SLE (2.6%) with PAH [25]. The Leal et al. study detected a higher prevalence of PAH as they diagnosed PH in 4 (11%) c-SLE cases [12]. PAH is one of the leading causes of mortality in adult SLE due to late symptom detection [35, 36]. PAH has several pathophysiologic mechanisms in SLE, such as thromboembolic diseases, including antiphospholipid antibody syndrome, non-inflammatory vascular remodeling, or pulmonary vasculitis. Early onset of PAH is most likely due to vasculitis with highly active SLE, which is reversible with intense immunosuppressive [37].

Cardiovascular implications in SLE are the sequelae of mixed pathophysiologic mechanisms, leading to the development of cardiac manifestations at a younger age than the general population. Endothelial cell dysfunction, disrupted innate immune system, and abnormalities in the adaptive immune system are incriminating factors in SLE-induced cardiac involvement. In addition, other SLE-related factors affect myocardial function, such as lupus nephritis, prolonged corticosteroid use, dyslipidemia, and vitamin D deficiency [38–40], hence the need for early surveillance of myocardial function in SLE patients.

Using conventional ECHO and tissue Doppler, the RV Tei index was significantly increased in patients compared to controls in the current work. Similarly, a study of RV functions in c-SLE documented higher tissue-Doppler-measured Tie index and s-wave velocity at tricuspid annulus was significantly lower in cases than in the control group [12]. In the current work, we had matching results; although they documented a significant increase of RV diameter using conventional ECHO that we did not encounter in our cohort.

Despite the non-significance of conventional LV FS and EF, Gin et al. found a significant decrease in systolic velocity of RV tissue Doppler in adult patients with SLE than in controls, especially in SLE patients with pulmonary hypertension. Additionally, the RV Tie index was higher in the SLE group with a statistically significant result [41]. Consistent findings were detected in the current work.

With increasing awareness of the RV’s role and the prognostic value in several cardiac diseases, a comprehensive, accurate evaluation of the RV is essential. Conventional ECHO for assessing RV function has inherent limitations because of the complex RV geometry and because most of these parameters depend on the angle or load. These issues were overcome with 3DE and deformation imaging with speckle-tracking ECHO (STE). Accuracy and reproducibility were proven relative to other reference procedures, such as radionuclide ventriculography and cMRI. However, it was reported that the duration of RV analysis (acquisition and offline reconstruction) with 3-DE is reasonably short, with satisfactory image quality [42–44].

TAPSE is an early-affected myocardial parameter in variable diseases [45, 46]. TAPSE represents longitudinal systolic function usually acquired by M-mode. It has been proven as a validated marker for RV systolic dysfunction. TAPSE has been validated to correlate strongly with RVEF measured by radionuclide angiography in adults [47]. This correlation was also replicated in the current work using Auto RV-derived TAPSE and RVEF. The TAPSE in our c-SLE patients was significantly lower than in the healthy volunteers. Furthermore, the mean Auto RV-FAC in our SLE cases was significantly lower than in controls, although it was within normal. It has been shown that RV FAC correlates with RV EF measured by cMRI [48]. RV FAC was more strongly correlated with the cMRI- derived RVEF better than the TAPSE in adults with PH [49].

3D-derived strains, including RVLS-free wall and RVLS-septum, were significantly affected in our c-SLE cases compared to healthy children. 3D RV lateral wall strain was defined as a critical parameter of RV dysfunction associated with SDI in the adult population independently of disease duration [18]. A study reported that RV peak longitudinal systolic strain was significantly reduced using 2D speckle tracking in c-SLE patients. This finding did not change after excluding patients with PH [12], which confirms that RV myocardial compromise in c-SLE cannot be exclusively attributed to pressure overload, validating the results on the SLE adult population [50]. Furthermore, Luo et al. evaluated RV strain using 2D STE in the adult population with SLE. They concluded that strain could early detect RV dysfunction in SLE patients with PAH, especially with mild PAH, which could guide early therapy, improving the prognosis and the quality of life of SLE patients with PH [13].

In the current work, 3D-derived RVEF and stroke volume were significantly decreased in c-SLE patients compared to the control group. Consistent results were reported by Buonauro and colleagues in uncomplicated SLE adult patients [18]. Nagata et al. compared RVEF values obtained by 3DE and cMRI and reported that RV EDV, ESV, and EF had good associations with those by cMRI (*r* = 0.74–0.9). In addition, 3DE-determined RV EF was independently associated with cardiac outcomes in patients with diverse backgrounds and offered incremental value over clinical risk factors and the other ECHO parameters for predicting future adverse outcomes [51]. However, it was reported that 3DE RV volumes calculated via 3DE tend to be significantly underestimated in comparison to cMRI [52, 53]. While fully automated 3DE RV quantification underestimated RV volumes, it successfully approximated RVEF compared to cMRI [54]. Regardless of these limitations, Auto-RV quantification estimate of RV function strongly correlated with right heart catheterization hemodynamic evaluation in PAH patients with high image quality [55]. Moreover, 3DE RV volumetric and EF measurements could be helpful in the assessment of early RV dilation or dysfunction that is not detectable using conventional ECHO with high reproducibility and accuracy [42, 56]. Correspondingly to other studies, excellent intra-and inter-observer ICC was evident for RV-derived EF in the current work.

One of the advantages of the fully automated software for 3D evaluation of RV is allowing for single-beat full-volume analysis with a satisfactory frame rate without stitching, making it easier to analyze patients with arrhythmias or those unable to hold breathing [57]. Therefore, we did not encounter problems with breathing maneuvers. Other older software requires multi-beat analysis with possible stitching effects with breathing.

No association was reported in the current work between all ECHO findings and specific clinical features of the diseases. However, Leal et al. reported an association between asymptomatic RV dysfunction, neuropsychiatric manifestations, and antiphospholipid antibodies in 35 children with SLE suggesting a common pathological pathway [12]. In addition, a recent study including 41 healthy control, 37 patients with extra-renal SLE, and 73 patients with lupus nephritis (LN) reported that patients with LN have more severe myocardial involvement than patients with extra-renal SLE in patients with LN, 24-h proteinuria was coupled with LVMD [58].

The RV evaluation studies in other pediatric rheumatological disorders are scarce. RV systolic and diastolic functions were reported to be impaired in children with juvenile idiopathic arthritis (JIA) compared to healthy controls but with no correlation of ECHO parameters to clinical manifestations of the disease [59], consistent with our findings in the current c-SLE cohort. Several mechanisms have been proposed for diastolic impairment in adults with rheumatoid arthritis (RA) and children with JIA, including fibrous scarring of the heart muscle, myocarditis, arteritis, nodular granulomatosis, amyloidosis, and cardiotoxic therapies [60].

The major limitation of the current research was that the study was a single-center experience with a non-blinding technique of the investigator regarding the study aim when analyzing the ECHO loops. Moreover, the inherent known limitation of 3DE is the need for good image quality at transthoracic ECHO full-volume dataset acquisition poses a slight risk of selection bias. Another limitation was the lack of concomitant transcatheter invasive pulmonary artery pressure and resistance measurements. Therefore, a large-scale multi-center study on a significant sample of c-SLE patients with variable ethnic backgrounds is warranted to confirm further the current study results. In addition, more extensive multi-center research might impact recommendations for the c-SLE routine follow-up process to expand to functional cardiac monitoring, including RV myocardial functions. Moreover, studying RV functions in c-SLE using cMRI could be the gold standard in future studies.

## Conclusion

Subtle right ventricular myocardial dysfunctional alterations could be detected in childhood-onset SLE patients, particularly via 3D auto RV-derived echocardiographic parameters, despite the absence of evident pulmonary hypertension. Furthermore, these parameters strongly correlate with the SLE disease activity and chronicity parameters such as damage index and cumulative SLEDAI score. Therefore, it is reasonable to consider including cardiac functional screening and RV in c-SLE cases.

## Data Availability

Relevant, de-identified data can be made available on request.

## Abbreviations

ACR: American College of Rheumatology
BMI: Body mass index
c-SLE: Childhood-onset systemic lupus erythematosus
cMRI: Cardiac magnetic resonance
ECHO: Echocardiography
EDV: End diastolic volume
EF: Ejection fraction
eGFR: Estimated glomerular filtration rate
FAC: Fractional area change
HMACQ: Heart Model Acquisition mode (HM ACQ)
IVCT: Isovolumic contraction time
LN: Lupus nephritis
LS: Longitudinal strain
LV: Left ventricle
LVPWD: LV posterior wall diameter
MMF: Mycophenolate mofetil
PAP: Pulmonary artery pressure
PH: Pulmonary hypertension
PAH: Pulmonary arterial hypertension
RAP: Right atrial pressure
RV: Right ventricle
RVAW: Right ventricle anterior wall
RVLS: RV lateral longitudinal strain
RVID: Right ventricle internal diameter
SLE: Systemic lupus erythematosus
SLEDAI: Systemic lupus erythematosus disease activity index
SDI: SLE International Collaborating Clinics/American College of Rheumatology Damage Index
SV: Stoke volume
TAPSE: Tricuspid annular plane systolic excursion
TRV: Tricuspid regurgitation velocity
3DE: Three-dimensional echocardiography
